# EEG CORRELATES OF SOMATIC DISORDERS IN DEPRESSIVE PATIENTS WHO SURVIVED AND HAVE NOT BEEN ILL WITH COVID-19

**DOI:** 10.1192/j.eurpsy.2023.1640

**Published:** 2023-07-19

**Authors:** A. F. Iznak, E. V. Iznak, E. V. Damyanovich

**Affiliations:** 1 Laboratory of Neurophysiology, Mental Health Research Centre, Moscow, Russian Federation

## Abstract

**Introduction:**

Coronavirus infection is accompanied by the development of a wide range of neuropsychiatric and somatic complications.

**Objectives:**

The aim of the study is to assess the severity of somatic disorders and to identify their EEG correlates in depressive patients who had and did not have COVID-19.

**Methods:**

The study involved 30 female depressive patients (F31.3-4, F21.3-4 + F34.0, according to ICD-10), aged 16-25 years, who previously had a mild or asymptomatic coronavirus infection (group "COVID”), and 40 depressive patients matched in gender, age and syndrome structure to patients of the “COVID” group, but who did not have COVID-19 (“non-COVID” group). The pre-treatment severity of depressive symptoms was assessed by the total sum, and by sums of clusters: depression (items 1, 2, 3, 7, 8), anxiety (items 9, 10, 11), sleep disorders (items 4, 5 , 6) and somatic disorders (items 12, 13, 14) of HDRS-17 scale. All patients underwent pre-treatment multichannel (16 leads) recordings of the background EEG followed by analysis of the absolute EEG spectral power (SP) in 8 narrow frequency sub-bands. Statistical analysis of the data was carried out using the methods of descriptive statistics and correlation analysis of the IBM SPSS Statistics, v.22 software package.

**Results:**

The values of the total sums of scores of individual clusters (depression, anxiety, sleep disorders) of the HDRS-17 scale in the “COVID” and “non-COVID” groups did not differ statistically. The exception was a significantly higher (p>0.01) number of complaints of somatic disorders (weakness, heaviness and pain in the muscles, a feeling of loss of energy, loss of strength, decreased libido) in patients who had COVID compared to those who did not (2.4±1.0 and 1.4±1.1 points, respectively). In the “non-COVID” group, the HDRS-17 somatic disorder cluster scores positively correlated with SP values of beta2 EEG (20–30 Hz) in leads F3, F8, and P3. which reflects the increased activation of brain stem structures, characteristic for depressive conditions. In the “COVID” group, these scores correlated with the SP values of alpha3 (11-13 Hz, in leads F4, F8, C4 and T4) and beta2 (20-30 Hz, in C4) not positively, but negatively. Thus, the severity of somatic complaints in patients of this group is associated not with greater, but with less activation of the brain (in particular, of the right hemisphere), which, presumably, may be associated with the “exhaustion” of the central mechanisms of regulation of autonomic functions after suffering COVID disease.

**Conclusions:**

COVID (in a mild or asymptomatic form) did not show a significant effect on the overall severity of depression of the studied group of patients who recovered from COVID, with the exception of a significantly greater severity of their somatic complaints compared to the group of patients who had not been ill with COVID. The study supported by the RSF grant No. 21-18-00129.

**Disclosure of Interest:**

None Declared

